# Perception of hypotheticality in technology-based business ideas: effects on Opportunity Beliefs from a Construal Level Theory perspective

**DOI:** 10.3389/fpsyg.2024.1404726

**Published:** 2024-06-07

**Authors:** Nelson A. Andrade-Valbuena, S. Sergio Olavarrieta, C. Juan Pablo Torres

**Affiliations:** ^1^Faculty of Economic and Administrative Sciences, Universidad Católica de la Santísima Concepción, Concepción, Chile; ^2^School of Economics and Business, Universidad de Chile, Santiago, Chile; ^3^School of Business, Universidad Adolfo Ibáñez, Santiago, Chile

**Keywords:** Construal Level Theory, hypotheticality, technology-based business ideas, Opportunity Beliefs, perception of technological innovation, psychological distance

## Abstract

This research investigates how entrepreneurs perceive the hypothetical nature of technologies (based on situations that are often imagined or theoretical) as a foundation for entrepreneurial endeavors and how this perception influences the formation of business Opportunity Beliefs. Drawing on the Construal Level Theory, we explore the relationship between the perceived hypotheticality of technologies and Opportunity Beliefs. Two experimental studies are conducted to examine these relationships, with Study 1 (*n* = 177 entrepreneurs) focusing on the perception of innovative technologies as more distant or hypothetical, and Study 2 (*n* = 404 entrepreneurs) delving into how the perceived distance to technology influences Opportunity Beliefs. The results indicate that entrepreneurs view more innovative technologies as more hypothetical and that hypotheticality mediates the relationship between the perceived degree of innovation and Opportunity Beliefs. We find evidence that Entrepreneurs tend to view the feasibility and fit/alignment of business opportunities more favorably when they perceive the psychological distance (hypotheticality) of the opportunity as closer rather than more distant. However, the difference this difference is nonsignificant in how they evaluate the desirability of the opportunity in any psychological distance. These results provide insight into the cognitive processes of entrepreneurs and offer implications for understanding how entrepreneurs perceive and evaluate business opportunities.

## 1 Introduction

During the early stages of entrepreneurship, a critical aspect involves evaluating opportunities (Shane and Venkataraman, 2000; Kuratko et al., 2021). This activity reflects a cognitive effort that entrepreneurs undertake (Baron and Tang, 2011; Tang et al., 2023), actively shaping their ideas about the interaction between demand and supply within an opportunity and the strategies to effectively leverage it in the marketplace (Gruber et al., 2015; Gielnik et al., 2018; Pollack et al., 2023). The evaluation of business ideas regarding their feasibility, desirability, and fit of the envisioned value proposition with market needs or problems it addresses has been explored in the evaluation of opportunities research as Opportunity Beliefs (Grégoire et al., 2009; Bocken et al., 2022). The pivotal role of these beliefs lies in their profound impact on the decision to embark on entrepreneurial endeavors (McMullen and Shepherd, 2006; Bergmann, 2017; Pollack et al., 2023), fostering heightened initiative and elevating the likelihood of successfully establishing a new business (Dimov, 2011; Nair et al., 2022). Consequently, unraveling the intricate mechanisms and influential factors that mold Opportunity Beliefs becomes paramount, offering a key avenue for advancing our comprehension of entrepreneurial behaviors and their subsequent outcomes.

Opportunity beliefs involve evaluating business ideas, which are perceptions about hypothetical scenarios or imaginary combinations of product or service offerings, target markets, and methods for bringing the offering into existence, along with projections about potential profits if it materializes (Shane and Venkataraman, 2000). This categorization of business ideas as events that could potentially happen but have not yet occurred places them in the realm of ‘what if’ scenarios, where entrepreneurs contemplate potential future outcomes or different circumstances (Grégoire et al., 2009; Davidsson, 2015). Prospecting a new product, a new client, or a new market presents unique situations, contexts, and dynamics that are subject to randomness in terms of their realization—where, when, and by whom they might occur (Alvarez et al., 2012), and therefore, with hypotheticality (Liberman and Trope, 1998). Hypotheticality, when viewed through a new business lens, refers to the extent to which an entrepreneurial event or situation is perceived as ‘distant’ or abstract from reality, with the ‘self’ serving as the reference point (Chen et al., 2018). This perception of distance also encompasses the perceived degree of separation in temporal (when), spatial (where), and social (to whom) aspects related to such events or objects from the entrepreneur (Liberman and Trope, 2008). Such distances are referred to as psychological distance (Liberman and Trope, 2014).

Analyzing Opportunity Beliefs about hypothetical situations becomes complex because individuals interpreting such scenarios may not do so equally (Dimov, 2011). Even a single individual, when imagining and prospecting business ideas at different times, might perceive them differently (McMullen and Shepherd, 2006; Van Gelderen et al., 2015). A classic example illustrating these differences is the rivalry between Blockbuster and Netflix in the video entertainment industry (Davis and Higgins, 2013). Blockbuster’s CEO, John Antioco, failed to recognize the potential of online streaming and rejected a partnership with Netflix. During this period, the market was shifting from physical rental stores to digital platforms due to advancements in internet technology and increasing consumer demand for convenience. Antioco’s decision was influenced by the prevailing business model of renting physical DVDs, which seemed successful at the time but was becoming outdated. In contrast, Netflix’s CEO, Reed Hastings, embraced the emerging online business model and streaming technology. Hastings recognized the increasing bandwidth of home internet connections and the growing popularity of on-demand content. By shifting Netflix from a DVD rental-by-mail service to an online streaming platform, he positioned the company to capitalize on these technological and market changes, leading to Blockbuster’s decline and Netflix’s success. While Antioco underestimated the significance of these technological shifts and the potential partnership with Netflix, Hastings interpreted these changes as a critical opportunity, which ultimately led to Netflix’s success. Why such differences? In essence, the factors contributing to these differing evaluations about opportunities remain inadequately understood, highlighting a crucial gap in understanding the implicit connection between entrepreneurial perception and evaluation.

While entrepreneurship research has highlighted the role of traits like experience, knowledge (Uygur and Kim, 2016; Wood et al., 2017), risk propensities (Forlani and Mullins, 2000; Mullins and Forlani, 2005), and heuristics and biases in shaping entrepreneurs’ judgments about business opportunities (Simon et al., 2000), there has been a notable gap in understanding the significance of individuals’ subjective perceptions. Existing literature also suggests that factors such as perceived familiarity of stimuli (Bhattacherjee and Premkumar, 2004), the degree of change or novelty (Sääksjärvi and Hellén, 2019), interpretations of complexity (Karahanna et al., 1999), and uncertainty about potential outcomes (Hu and Reid, 2018) influence the formation of Opportunity Beliefs. However, limited attention has been paid to exploring how varying degrees of innovation in venture ideas impact entrepreneurs’ beliefs, particularly in the context of innovative technologies. This gap warrants further exploration to understand how these innovations shape entrepreneurs’ assessments of their potential for generating economic rents.

This study seeks to investigate how entrepreneurs perceive the hypothetical nature of technologies as a basis for entrepreneurial endeavors, particularly when these technologies serve as stimuli, and how this perception influences the formation of business Opportunity Beliefs. Highly hypothetical events or situations are those considered improbable or purely speculative, while less hypothetical ones are deemed more likely or carry a higher perceived probability of occurrence (Tumasjan et al., 2013; Chen et al., 2018). To explore this distinction, we draw on insights from social psychology, specifically Construal Level Theory (CLT), which examines variations in perception, interpretation, and evaluation (Trope et al., 2007). According to CLT, individuals mentally construe things differently based on their psychological distance from themselves (Liberman et al., 2002). When events are psychologically distant, people tend to contemplate them in a more abstract, high-level, or global manner (Liberman et al., 2007). Conversely, when events are psychologically close, they are perceived in a more concrete, low-level, or detailed way. Since different levels of abstraction can significantly influence how individuals perceive and make sense of the world, these insights have implications for their evaluations and judgments (Trope et al., 2007).

This study seeks to address two primary questions: First, whether entrepreneurs perceive more innovative technologies as being more hypothetical. Second, if so, how the perception of innovativeness and hypotheticality of technologies influences overall Opportunity Beliefs. We propose that the influence of hypotheticality affects entrepreneurs’ belief in opportunities, with hypotheticality playing a mediating role in the connection between the perception of the degree of innovation and an entrepreneur’s belief in opportunities. This influence is reflected in the evaluation of the favorability of overall business Opportunity Beliefs, which, in turn, impacts the different dimensions of these beliefs: Feasibility and Desirability of the business idea, and its Fit/Alignment with the market.

The presented line of thought is tested through two studies, each involving different sets of technologies. Study 1 examines whether entrepreneurs perceive more innovative technologies as psychologically more distant, or hypothetical compared to less innovative ones. The results confirm that entrepreneurs indeed view more innovative technologies as more distal or hypothetical. In Study 2, we explore how the formation of business Opportunity Beliefs is influenced by the psychological distance to technology, which is shaped by the perceived level of innovation. The results indicate that an individual’s subjective sense of distance (hypotheticality) serves as a mediator in the relationship between their perception of the degree of innovation in technologies and their Opportunity Beliefs. Our findings suggest that when entrepreneurs assess their overall Opportunity Beliefs, they tend to arrive at more favorable outcomes when technologies are perceived as less hypothetical. However, when examining specific aspects of Opportunity Beliefs, entrepreneurs assess the desirability of technological advancements as a foundation for entrepreneurial pursuits similarly, regardless of whether they are perceived as hypothetical or not. Furthermore, our research reveals that when technologies are seen as less hypothetical, entrepreneurs tend to perceive businesses built upon these technologies as more feasible and better suited to the market. They also perceive them as more effective at addressing customer problems and needs compared to when technologies are seen as more hypothetical.

In the following sections, we elaborate on the connections between Opportunity Beliefs and Hypotheticality, focusing specifically on hypotheticality regarding technology-based business ideas, which are central elements of our theoretical framework. We then outline our hypotheses based on Construal Level Theory (CLT). Next, we provide a summary of the Methods, Data Analysis, and findings from both Study 1 and Study 2, followed by a detailed discussion of these results. Finally, we conclude with an extensive discussion section that addresses theoretical and empirical implications, along with proposing a future research agenda to further develop Opportunity Beliefs research.

## 2 Conceptual background

### 2.1 Opportunity beliefs, business ideas, and hypotheticality in the first stages of entrepreneurial endeavors

The Opportunity Beliefs concept builds on theories about the evaluation of business ideas and entrepreneurial action (McMullen and Shepherd, 2006). From this perspective, individuals undertake actions based on their desires, assumptions, and interpretations of their surroundings, their own positions, the positions of other actors within that context, and the anticipated outcomes of everyone’s actions (Hastie, 2003; Wood et al., 2014; Williams and Wood, 2015). This subjective process, in turn, affects individuals’ attitudes toward the entrepreneurial endeavor, shaping their beliefs about the potential value of exploiting those opportunities (Baron and Ensley, 2006; Mitchell et al., 2017). As a result, entrepreneurial endeavors stem from individuals’ thoughts and beliefs that, given their perceptions and comprehension of a particular scenario, introducing a new product or service presents appealing and viable entrepreneurial prospects, while also assessing their alignment with market demands (Grégoire and Shepherd, 2012).

Ontologically, Opportunity Beliefs differ from the entrepreneurial intention or decision to act on or exploit a business idea (Bergmann, 2017; McMullen and Kier, 2017), which involve concepts such as First- and Third-person opportunities developed around notions of Feasibility (the required knowledge and skills related to the business) and Desirability (motivations for entrepreneurial action) (Mitchell and Shepherd, 2016). In this vein, Opportunity Beliefs also distinguish themselves from Opportunity Creation, which, based on design principles (Simon et al., 2000), emphasizes entrepreneurs’ focus on taking action trough bricolage by utilizing available resources to craft an opportunity (c.f., Baker and Nelson, 2005; Miller, 2007; Dimov, 2011; Baier-Fuentes et al., 2023). Opportunity Beliefs also have an ontological difference from the concept of Opportunity Recognition, which posits that opportunities present objective factors that do not exist solely in the minds of entrepreneurs, implying that opportunities, in some sense, exist (Eckhardt and Shane, 2003). Opportunity Recognition suggests that certain individuals are more Alert at ‘identifying’ opportunities at the outset of their entrepreneurial endeavors, while others may not be (Shane and Venkataraman, 2000; Tang et al., 2012). Furthermore, Opportunity Beliefs differ from other concepts that focus on the feasibility and desirability of business ideas in other studies (e.g., Edelman and Yli-Renko, 2010; Tumasjan et al., 2013), as well as concepts that revolve around prospects of gains and losses associated with these ideas (e.g., Shepherd and DeTienne, 2005; Grichnik et al., 2010), which capture distinguishable phases in the assessment process (e.g., Shepherd et al., 2007; Welpe et al., 2012; Tumasjan et al., 2013; Wood and Williams, 2014).

Opportunity Beliefs are rooted in the perceptions, interpretations, and attributes assigned to business ideas as stimuli, ultimately influencing individuals’ judgments concerning the extent to which these ideas represent a desirable and feasible entrepreneurial path (Grégoire and Shepherd, 2012; Wood and Mckelvie, 2015). Opportunity Beliefs can be defined as an individual’s confidence in the viability of a business opportunity (Grégoire et al., 2009). This construct encompasses three dimensions (c.f., Baron and Ensley, 2006; Stevenson and Carlos Jarillo, 2007; Grégoire and Shepherd, 2012; Tumasjan et al., 2013; Mitchell and Shepherd, 2016): Feasibility summarizes the perceived difficulty, efforts, and resources required to obtain such a reward (i.e., the necessary knowledge, skills, and abilities). Desirability reflects the perceived usefulness and value of the opportunity, addressing what the entrepreneur stands to gain from it (i.e., potential rewards). The difference between the two can be viewed as the distinction between means (answering the “how” aspects of the opportunity) and ends (answering the “why” aspects of the opportunity) associated with their prospective exploration and exploitation (Tumasjan et al., 2013). Fit/Alignment, stemming from the introduction of new means/ends relationships, denotes qualities that align with the needs and requirements of a target market (Grégoire et al., 2009; Grégoire and Shepherd, 2012). In this context, the concept of Opportunity Beliefs shares similarities with Opportunity Feasibility Belief (Dimov, 2011), Perceived Market Opportunity (Edelman and Yli-Renko, 2010), and Opportunity Confidence (Davidsson, 2015).

Opportunity beliefs revolve around entrepreneurs’ primary task of creating and/or evaluating hypotheses concerning the relationship between the demand side (such as market desires or needs) and the supply side (like a novel product, service, technology, or business model) of an opportunity (Grégoire and Shepherd, 2012). Opportunity Beliefs take shape through a cognitive process in which individuals make prospections regarding the economic feasibility and market compatibility of a business idea (Grégoire et al., 2009). Such prospects depend on the interpretation of the idea itself (whether it originates from oneself or from others) and on the circumstances within the business environment, which begin at the aggregate level and have the potential to elicit or facilitate the new venture process (Davidsson, 2015). This process stimulates the subjective attribution of traits to either the business idea or the circumstances, thereby shaping individuals’ perspectives and attitudes toward them (Mitchell et al., 2017). External elements, such as groundbreaking technologies, shifts in the landscape of scientific knowledge, and transformations in technology itself, serve as concrete examples of environmental changes capable of consistently altering the perceived value of business ideas (c.f., Schumpeter, 1934; Kirzner, 1979). Such changes within the business environment have been referred to in the entrepreneurship literature as “External Enablers” (Davidsson et al., 2022). In essence, the interplay between Opportunity Beliefs and External Enablers underscores how cognitive processes and environmental dynamics collectively contribute to the formation and evolution of the evaluation of entrepreneurial opportunities.

External Enablers, when viewed as contextual factors, have been studied through the lenses of their individuals’ familiarity (Bhattacherjee and Premkumar, 2004), the perceived difficulty linked to their use (Davidsson et al., 2022), the ambiguity surrounding their resultant effects (Karahanna et al., 1999), their innovative nature, adaptability, or adaptational potential (Cassar, 2014), and their feasibility for utilization (Uygur and Kim, 2016). In particular, studies examining technological factors as stimulus for new venture ideas have investigated how inventions, novel products, and technologies (e.g., Simon et al., 2000; Shepherd and DeTienne, 2005) and specific elements within them (e.g., McMullen and Shepherd, 2006; Grégoire and Shepherd, 2012) trigger, prompt, or hinder cognitive processes (e.g., Corbett, 2007; Autio et al., 2013; Kier and McMullen, 2018) that lay the groundwork for the development of new economic endeavors. This suggests that many critical considerations related to new venture ideas, such as customer problems and needs (Shane, 2000; Shepherd and DeTienne, 2005; Tang et al., 2012), the suitability of products and services to meet these requirements (Lee and Venkataraman, 2006), and prospects for gains and rewards (Mullins and Forlani, 2005; Welpe et al., 2012), are also influenced by perceptions and characteristics ascribed to the stimulus.

External Enablers, whether they act as facilitating circumstances or catalysts for new venture outcomes, inherently necessitate a cognitive assessment of their hypothetical nature concerning their ability to stimulate or facilitate the potential exploitation of the underlying business idea (Chen et al., 2018). This is clearly exemplified in technological developments. For instance, new technologies, especially those at the cutting edge of advancement, often exist more as conceptual or theoretical elaborations rather than fully developed and tangible products or solutions (Choi et al., 2022; Ho, 2022), implying a certain level of hypotheticality. Similarly, whether a new technology will be widely accepted by consumers, businesses, or industries is often hypothetical, as the success of the innovation is not guaranteed (Steiber et al., 2020; Jiang et al., 2021). People may be uncertain about their feasibility (Moghavvemi et al., 2016), the timeline for their full development (Jabeur et al., 2014), their dependence on enabling technologies (Nylund et al., 2022), and the availability of complementary goods (Cenamor and Frishammar, 2021), as well as their potential impacts (Hall et al., 2014). This uncertainty contributes to their hypothetical nature, as they are viewed as future possibilities with unknown outcomes.

Building upon the overall framework, we will now develop hypotheses concerning the psychological distance from technologies when they serve as External Enablers and their influence on Opportunity Beliefs. Specifically, our focus is on hypotheticality as a dimension of psychological distance which is derived from the level of innovation in the technology, within the framework of the Construal Level Theory (CLT).

### 2.2 Hypothesis development

#### 2.2.1 Technological developments and psychological distance

Psychological distance refers to the perceived degree of separation of an individual across four dimensions: temporal (when), spatial (where), social (to whom), and hypotheticality (the likelihood of occurrence) (Liberman and Trope, 1998). Temporal distance signifies the time gap between the perceiver’s “now” and a specific event (Liberman and Trope, 2008). Spatial distance reflects the physical gap between the perceiver’s “here” and an event or object (Liberman et al., 2012). Social distance gauges the level of familiarity or understanding from the perceiver’s perspective (Liberman and Trope, 2014). Hypothetical distance quantifies how real or imaginary an action, person, or object can be perceived (Trope et al., 2007). In essence, psychological distance is anchored to the self as a reference point, representing a subjective experience of the perceived remoteness or proximity between an object or event and the individual (Liberman and Trope, 1998).

External factors that emerge at an aggregate level, such as technological breakthroughs, shifts in scientific knowledge, and advancements in technologies, have the potential to initiate and reshape outcomes across a diverse range of new economic activities (Schumpeter, 1934; Kirzner, 1973). These events, along with the extent of changes they could bring to resulting products (Linton, 2002), represent distinct situations, contexts, and dynamics characterized by randomness in terms of where, when, and by whom they will be realized (Alvarez et al., 2012). These conditions are often perceived as unpredictable influences and/or facilitators of entrepreneurial endeavors (Kimjeon and Davidsson, 2022). This unpredictability may be associated with hypotheticality and, as a result, with how psychologically distant they are perceived due to their uncertain nature (Mount et al., 2021).

Hypotheticality also relates to the level of abstraction or precision in how individuals mentally represent events or scenarios (Smith and Trope, 2006). Since new technological breakthroughs often involve advanced scientific principles and theoretical possibilities, this can contribute to their hypothetical distance from the self, as they have not yet been fully realized or understood in practical terms (Chiang et al., 2022). In this context, people may also harbor uncertainties about the feasibility, utility, or potential impact of these unknown technologies, which further increases psychological distance (c.f., Liberman and Trope, 1998). Additionally, one of the underlying principles behind the emergence of new technological products as catalysts for new business opportunities is that they elicit qualities of rarity, novelty, and change (Shane and Venkataraman, 2000). The introduction of novel functionalities and improvements when compared to existing alternatives might play a significant role in how they are perceived in terms of distance, as they generate perceptions of familiarity and experience (Bhattacherjee and Premkumar, 2004).

Considering the factors discussed above and aligning with CLT, the degree of innovativeness in technologies may result in perceptions of unfamiliarity, unknowability, or inexperience. According to CLT, these perceptions might induce psychological distance from the observer’s perspective (c.f., Trope et al., 2007). This leads to the following hypothesis:

*H1*: Technologies perceived as more innovative (vs. less innovative) by entrepreneurs, will be sensed as more (vs. less) psychologically distant.

#### 2.2.2 Psychological distance and business opportunity beliefs from technological developments

The development of beliefs regarding business opportunities involves an initial phase of cognitive processes that center around business ideas, perceiving events, or external conditions as stimuli (Kimjeon and Davidsson, 2022). Particularly, when analyzing the use of any technology as a foundation for entrepreneurial endeavors, individuals are first engaged in a reflective process driven by these stimuli, serving as a new framework of information (Andrade-Valbuena and Torres, 2018). To form convictions about how feasible it is to utilize the technology or to gain a deeper understanding of it, individuals might shift their perception from a broad and theoretical standpoint to a more concrete cognitive framework based on analogous firsthand experiences (Venkatesh and Davis, 2000). This process involves drawing insights from personal experiences or perspectives, as well as evaluating and contemplating the personal relevance and practicality of its adoption to the self, and to others (Schweitzer et al., 2015).

The developmental context of Opportunity Beliefs encompasses a second phase involving the creation and evaluation of assumptions concerning the connection between the demand and supply aspects of an opportunity and how individuals can gain advantages from its exploitation (McMullen and Shepherd, 2006). In this context, research on Construal Level Theory (CLT) has shown that as psychological distance increases, objects or events are perceived with a more abstract, general, and fundamental perspective (Smith and Trope, 2006). This shift entails a reduction in specific, unique, and peripheral details but also results in the attribution of new interpretations (Trope et al., 2007). These alterations in representation have an impact on assessments, predictions, and preferences (Liberman et al., 2007). For instance, higher levels of construals stemming from the hypotheticality associated with technological innovations in relation to Opportunity Beliefs might be linked to market uncertainties, as the acceptance of these innovations by consumers, businesses, or industries is not guaranteed (Linton, 2002; Chiang et al., 2022). In this regard, greater psychological distance may lead to more abstract and high-level construals of the business opportunity (Chen et al., 2018). These high-level construals can impact Opportunity Beliefs, as they are associated with a more distant, big-picture view of the opportunity’s potential (Mount et al., 2021). In this context, more innovative technological-based business ideas might be perceived as more hypothetical, challenging to implement, and less immediate due to their abstract and future-oriented nature, which contributes to greater psychological distance (Reyt et al., 2017). This effect implies that the degree of innovation indirectly affects Opportunity Beliefs through its influence on psychological distance. Thus:

*H2a*: Degree of innovation in technologies (External Enabler) affects psychological distance, which in turn affects Opportunity Beliefs, such that psychological distance would mediate the relationship between the degree of innovation and Opportunity Beliefs.

In the realm of business ideas characterized by varying degrees of technological innovation, the hypothetical nature can become so prominent that deliberations about these opportunities start to resemble scenarios akin to gambling (c.f., Barney, 1997; Danneels, 2004). For instance, in the case of ideas based on disruptive technologies, the probability of technological adoption and commercial success within a business context mirrors scenarios that are less credible, challenging to envision, and, in this respect, more hypothetical (Mount et al., 2021). Conversely, less innovative technologies in a business context give rise to scenarios that are often viewed as more concrete, which consequently, are more readily associated with tangible products or solutions (Akdim et al., 2023). This, in turn, naturally affects an individual’s assessment of the attractiveness of a business opportunity associated with such technologies. More precisely, higher probabilities of realization, stemming from less innovative technologies, are expected to enhance individuals’ overall Opportunity Beliefs. Thus:

*H2b*: Entrepreneurs will evaluate more favorable overall business Opportunity Beliefs when psychological distance (hypotheticality) is close, than when psychological distance (hypotheticality) is more distal.

#### 2.2.3 Psychological distance and its effects on the dimensions of business opportunity beliefs

According to CLT, psychological distance affects how individuals will process relationships or similarities between objects, events, and goals on different levels of mental abstractions (Liberman and Trope, 2014). At low levels of representation, the individual is focused on specific details, secondary and incidental features related to feasibility concerns, means to achieve the value of the end state, and how the event is performed (concrete processing) (Smith and Trope, 2006). In this sense, in cases when entrepreneurs are presented with business ideas that prime low level of hypotheticality (psychological distance is close, associated with lower perceptions of innovativeness of the business idea), CLT predicts that individuals tend to adopt a more concrete and detailed mindset (Liberman et al., 2002). They might focus on specific, practical aspects of the opportunity and consider it in a more immediate and straightforward manner. In this state of mind, entrepreneurs may perceive the feasibility dimension as manageable and the necessary efforts and resources as attainable (Duan et al., 2022). They are more likely to have a clear understanding of “how” rewards associated with the opportunity can be achieved, which might lead to a more favorable evaluation of feasibility (Ram et al., 2022).

In contrast, higher levels of conceptualization encourage several superordinate central features and general representations of the action related to desirability concerns, where the individual focuses on the value of the end state, and why the event is performed (abstract processing) (Tumasjan et al., 2013). In these situations, when entrepreneurs encounter business ideas characterized by a high degree of hypotheticality (indicating a distal psychological distance and associated with a perception of greater innovativeness in the business idea), CLT predicts that individuals tend to engage in more abstract and high-level thinking (Smith and Trope, 2006). They might approach the opportunity from a broader and more conceptual standpoint. As a result, they may perceive the feasibility dimension as more challenging, lacking a clear, step-by-step plan for obtaining the rewards (Duan et al., 2022). This distant perspective can make the efforts and resources required to achieve those rewards appear less accessible and more demanding, leading to a less favorable evaluation of Feasibility (Ram et al., 2022). Thus:

The above reasoning leads to propose the following hypothesis:

*H3*: Entrepreneurs will evaluate more favorable the Feasibility dimension of Opportunity Beliefs when psychological distance (hypotheticality) is near, than when psychological (hypotheticality) distance is more distal.

Applying a similar rationale than the above, when entrepreneurs are confronted with business ideas characterized by a diminished level of hypotheticality (indicative of proximity in psychological distance and connected to reduced perceptions of innovativeness within the business idea), CLT posits that individuals are inclined to adopt a more tangible and detailed perspective (Liberman and Trope, 2008). Within this cognitive state, their focus shifts toward the concrete and pragmatic aspects of the opportunity, fostering a more immediate and uncomplicated evaluation (c.f., Duan et al., 2022). In this frame of mind, entrepreneurs may perceive the degree of alignment between an opportunity’s specific means of supply and a target market, taking into consideration the qualities that cater to the needs and demands of that market, as an attainable goal. This, in turn, could lead to a more favorable assessment of Fit/Alignment (c.f., Liberman and Trope, 2008).

On the contrary, when entrepreneurs are presented with business ideas characterized by an amplified level of hypotheticality (indicative of greater psychological distance and associated with heightened perceptions of innovativeness within the business idea), CLT postulates that individuals tend to indulge in more abstract and high-level cognitive processes (Liberman and Trope, 1998). Consequently, they may assess the Fit/Alignment dimension from a broader and more conceptual standpoint. This broader evaluation might lead them to perceive that the qualities aligning with the needs and demands of the market are less readily attainable, ultimately resulting in a less favorable appraisal of Fit/Alignment (c.f., Mount et al., 2021). Thus:

*H4*: Entrepreneurs will evaluate more favorable the Fit/Alignment dimension of Opportunity Beliefs when psychological distance (hypotheticality) is near than when psychological distance (hypotheticality) is more distal.

In the domain of technology-driven business prospects, assessments of business ideas are often made with limited or no prior knowledge, indicating that judgments are made under low conditions of predictability (Alvarez and Barney, 2005). In such situations, tech-based business ideas that are more innovative are regarded as costlier and riskier, with lesser probability of occurrence (Odrakiewicz et al., 2012). Additionally, there are stronger beliefs and expectations of significant profits and the potential for the venture to serve as a major driver of economic growth if it succeeds (Pellikka et al., 2016). In the context of businesses ideas that rely on highly innovative technologies, the prospects of success are influenced, at least in part, by luck (Barney, 1997; Alvarez and Barney, 2005) and prospections about efforts and actions that need to be undertaken, as resource allocation and managing heightened uncertainty (Giones et al., 2013). In this regard (Todorov et al., 2007), in experimental contexts of analyses of judgments and monetary decisions demonstrated that when individuals anticipated low probabilities of occurrence, they might form their judgments about the attractiveness of an outcome, while ignoring the efforts and resources required for its realization. Conversely, in situations where individuals expected high probabilities of occurrence, both efforts and rewards are considered equally when evaluating choices.

Taking the above into consideration, when business ideas exhibit higher hypotheticality, indicating lower probabilities of occurrence, the evaluation of their Desirability becomes contingent on what potential entrepreneurs can gain and the efforts needed for implementation. Consequently, this may lead individuals to assess the Desirability of the business idea more critically. Conversely, when business ideas have lower hypotheticality, signifying higher probabilities of occurrence, the assessment of their Desirability could primarily revolve around the potential gains for entrepreneurs, diminishing the significance of the distinction between these two scenarios (high vs. low levels of hypotheticality). Thus:

*H5*: The difference in how entrepreneurs evaluate the Desirability dimension of Opportunity Beliefs when psychological distance is close compared to when it is more remote will be negligible.

## 3 Methods, data analysis, and results

In conducting two studies, we adopt an experimental approach embedded in online surveys to investigate Opportunity Beliefs. Manipulating information and stimuli is deemed the most suitable method for probing into Opportunity Beliefs (Grégoire and Shepherd, 2012). The random assignment of individuals to different conditions or treatments is employed, minimizing support for counterfactual inferences (Shadish, 2002). Given that contemplating new economic activities based on technologies necessitates exposing participants to stimuli for belief development, employing a control group becomes impractical due to this inherent exposure. Consequently, we employ a factorial-based experimental design featuring two levels of construal based on perceived innovation: high vs. low, representing more vs. less psychological distance (Study 1), and more vs. less hypothetical (Study 2). This design allows us to systematically vary the cognitive framing of stimuli, facilitating a nuanced exploration of the impact of psychological distance on Opportunity Beliefs.

Experimental stimuli were derived from two primary criteria: technologies that vary in perceived innovation within the same category and technologies with similar awareness levels among respondents. Initially, a diverse pool of technologies was collected by examining the webpages of renowned consulting firms. Following discussions with technology experts led to the elaboration of Vignettes based on two pairs of technologies and their respective categorizations: Ultra Smart Textiles “UST” (most innovative) and Waterproof Breathable Textiles “WBT” (less innovative) from the textile industry, and Affective Computing “AC” (most innovative) and Fingerprint Identification “FID” (less innovative) from the realm of human identification technologies.

Vignette-based experiments can raise concerns due to their artificial nature. Therefore, it is crucial to ensure their alignment with real-world situations (Gaglio and Katz, 2001). To achieve this, written descriptions were crafted to mirror concise tech trend reports that inform individuals about new technologies with potential business applications, akin to those consulted on various webpages. These descriptions conveyed details about the technology’s functionality, applications, and growth prospects. Multiple think-aloud tests were conducted with individuals of varying technological and entrepreneurial backgrounds to confirm face validity (see Vignettes in the Supplementary material).

### 3.1 Study 1: does technological innovation produce psychological distance effects in its regard?

The aim of Study 1 is to collect evidence regarding the perception that highly innovative technologies are often considered distant by nature, as proposed in Hypothesis 1. Study 1 also serves as a test of the suitability of the stimuli for use in the subsequent Study 2.

#### 3.1.1 Instruments

A three-item scale was adapted from Park et al. (2020), considering the hypothetical dimension of psychological distance adjusted in a differential semantic scale [i.e., for me, the technology described is more… (1) Real/(6) Fictional; (1) Unquestionable/(6) Questionable; (1) Factual/(6) Illusory].

A four-item scale adapted from Lowe and Alpert (2015) was used to evaluate the perceived level of innovation of the technology (i.e., how innovative is the selected technology, 1 = Not innovative to 6 = Very innovative).

Following (Gartner et al., 2004), two questions were used to ensure the inclusion of respondents with diverse levels of experience in starting new ventures. These questions asked respondents, either alone or with others, whether they had attempted to start a new business intended to be their primary source of income at any time (to ascertain the entrepreneurial status of respondents); and if so, the number of such businesses they had attempted to start.

Controlling potential confounding effects of psychological distance to technologies, a three-item scale of Technological Awareness, adapted from Schweitzer et al. (2014), was included (i.e., I am well-informed about new developments in this technology; 1 = Strongly disagree to 6 = Strongly agree).

#### 3.1.2 Data research topic and experimental procedure

Participants were recruited through Amazon MTurk, in alignment with previous research on entrepreneurship experiments and earlier calls for research on using online labor markets (Aguinis and Lawal, 2012; Gupta et al., 2014; Frederiks et al., 2019). Following best practices for MTurk environments (see 106–108), U.S. citizen respondents with an approval rate above 98% and a number of HITS approved greater than 500 were recruited for payment and were informed that the survey focused on their perception of technological products.

Respondents began the study evaluating the perception of sophistication of a list of technologies [(1) not sophisticated to six (6) very sophisticated] including focal technologies, and randomly assigned to one of the conditions provided with the description designed and asked to complete the measures designed. Control questions were asked to verify the ease of understanding of the reading, and to eliminate responses from speeders, producing flawed or misleading data, as suggested by Ford (2017). Finally, some demographic questions were added related to age, gender, and maximum studies achieved. This experimental procedure was followed also in Study 2.

#### 3.1.3 Results

##### 3.1.3.1 Participant characteristics

One hundred and seventy-seven (177) responses from entrepreneurs (60.45% male) with an average age of 38.71 years, of whom 72.30% had completed a college degree, were retained for analysis. On average, entrepreneurs have attempted to establish a new business intended to be their primary source of income, with an average of 4.04 attempts in the short term.

##### 3.1.3.2 Hypothesis testing

Comparing the median score for the Mann–Whitney *U*-test in the technologies presented, the population participating in the high-level technology condition rated the level of innovation perceived of the technology presented significantly higher. In the textile industry, (MdUST = 5.5, *n* = 41 vs. MdWBT = 4.0, *n* = 44; *U* = 463.0, *z* = −3.92, *p* < 0.001, *r* = 0.42) (*α* = 0.85) and in human identification technologies (MdFID = 3.75, *n* = 49 vs. MdAC = 5.25, *n* = 43; *U* = 639.5, *z* = −3.92, *p* < 0.001, *r* = 0.39) (*α* = 0.83). This was corroborated by results from a Kruskal-Wallis test of the high vs. low conditions [MdHigh = 4.0, *n* = 84 vs. MdLow = 2.75, *n* = 93; *χ*^2^(1, *N* = 177) = 16.88, *p* < 0.001], suggesting that the innovation-level manipulation between the stimuli was appropriate.

Prior to H1 testing, the median averaged score of technological awareness for each of the four stimuli was compared using the Mann–Whitney *U*-test. Results revealed that the participant population had an equal level of awareness of technologies in the textile industry (MdUST = 4.0, *n* = 41 vs. MdWBT = 4.0, *n* = 44; *U* = 1175.50, *z* = −0.00, *p* > 0.1) (*α* = 0.85) and in human identification technologies (MdFID = 4.00, *n* = 49 vs. MdAC = 4.00, *n* = 43; *U* = 1175.50, *z* = −0.04, *p* > 0.1) (*α* = 0.83). Therefore, the data across each condition were collapsed.

The results of a Mann–Whitney *U*-test showed that participants in the high-level of innovation condition (vs. low-level condition), perceived these technologies as being more psychologically distant (More hypothetical), as predicted in H1 [(MdHigh = 4.0, *n* = 84 vs. MdLow = 2.66, *n* = 93; *U* = 5288.00, *z* = −4.070, *p* < 0.001, *r* = 0.31) (*α* = 0.88)]. Thus, Hypothesis 1 was supported.

#### 3.1.4 Discussion of Study 1

The results of Study 1, which were replicated using two distinct sets of technologies, substantiate the proposition stated in Hypothesis 1 that more innovative technologies tend to be inherently perceived as distant. This confirmation serves as a foundational prerequisite for the subsequent study, as it underscores the idea that psychological distance plays a role in the formation of Opportunity Beliefs. Furthermore, these findings eliminate the potential influences of technological awareness, as there were no discernible distinctions among the four presented scenarios.

### 3.2 Study 2: effects of psychological distance (hypotheticality) on the formation of business opportunity beliefs

Study 2 seeks to empirically demonstrate how hypotheticality, a factor of psychological distance based on the level of technological innovation, shape the different dimensions of business Opportunity Beliefs (H2 to H5). As same as in Study 1, we consider two different sets of technologies attempting to validate our findings. Predictions in a single between-subjects factor (technology innovation: high vs. low) design were tested, utilizing the four technologies developed as stimuli in Study 1.

#### 3.2.1 Instruments

The three dimensions of business Opportunity Beliefs were assessed using the scales developed by Grégoire et al. (2009), employing a Likert scale ranging from “strongly disagree” (1) to “strongly agree” (6). Hypotheticality was gauged with a three-item scale adapted from Study 1. The same control measures utilized in Study 1, which included the evaluation of technological awareness (Schweitzer et al., 2014) and entrepreneurial experience (Adapted from 101), were also incorporated.

#### 3.2.2 Data research topic and experimental procedure

The experimental approach embedded in online surveys methodology used in Study 1 was replicated in this study. Participants were recruited for payment through Amazon MTurk, following best practices for MTurk environments (see Ford, 2017; Aguinis et al., 2020a,b). They began by assessing the perceived sophistication of a list of technologies, including focal technologies, using a scale from 1 (not sophisticated) to 6 (very sophisticated). Subsequently, they were randomly assigned to one of the conditions and asked to complete the specified measures.

#### 3.2.3 Results

##### 3.2.3.1 Participant characteristics

Four hundred and four (404) responses from entrepreneurs, of which 52.48% were male, with an average age of 40.99 years, and 76% having completed a college degree, were included in the analysis. On average, entrepreneurs have tried or attempted to establish a new business as their primary source of income in the short term, with an average of 3.72 new business endeavors.

##### 3.2.3.2 Hypothesis testing

Results from a Kruskal-Wallis test considering hypotheticality revealed that the manipulation of distance between the stimuli was appropriate, showing statistically significant difference in psychological distance across the high-low condition in the textile industry [(MdUST = 4.00, *n* = 81 vs. MdWBT = 3.00, *n* = 101; *χ*^2^(1, *N* = 182) = 18.067, *p* < 0.001), (*α* = 0.91)] and in the identification and human recognition technologies [(MdAC = 4.0, *n* = 112 vs. MdFID = 2.00, *n* = 110; *χ*^2^(1, *N* = 222) = 27.18, *p* < 0.001), (*α* = 0.88)].

###### 3.2.3.2.1 Effects of hypotheticality on overall business opportunity beliefs

The Sobel (1982) test was performed to evaluate significance of the mediation effects of Hypotheticality among the Degree of innovation in technologies (External Enabler) and Opportunity Beliefs (OB) (H2a). The values of the Sobel test of are above 1.96 and significant (Overall OB-Sobel test statistic: 7.23, *p* = 0.00; Textile industry OB-Sobel test statistic: 4.15, *p* = 0.00; Identification and human recognition technologies OB-Sobel test statistic: 4.31, *p* = 0.00). Hence, Psychological Distance (Hypotheticality) has mediation effects among the Degree of innovation in technologies (External Enabler) and Opportunity Beliefs (OB) thus, supporting H2a.

Prior to testing H2b, the median averaged scores of Opportunity Beliefs for each of the four stimuli was compared using the Mann–Whitney *U*-test. Results revealed that in the low condition of technologies, the participant population evaluated equally the Opportunity Beliefs [MdWBT = 4.72, *n* = 101 vs. MdFID = 4.66, *n* = 110; *U* = 5282.00, *z* = −0.62, *p* > 0.1, (*α* = 0.88)]. Similar results in the high condition of technologies were obtained [MdUST = 4.56, *n* = 81 vs. MdAC = 4.36, *n* = 112; *U* = 3992.00, *z* = −1.42, *p* > 0.1, (*α* = 0.92)]. Considering these results, the data were collapsed across each condition.

Regarding the general Opportunity Beliefs, respondents in the low condition (vs. high) of the level of psychological distance, evaluated overall business opportunities more favorably (MdGENERAL-High = 4.23, *n* = 193 vs. MdGENERAL-low = 4.72, *n* = 211; *F* (1,402) = 12.343, *p* < 0.001). These results are confirmed by a Kruskal-Wallis’s test pondering influences of high vs. low settings on General Opportunity Beliefs [*χ*^2^(1, *N* = 404) = 9.253, *p* = 0.002], thus supporting H2b.

###### 3.2.3.2.2 Effects of hypotheticality on the dimensions of opportunity beliefs

Prior to testing H2 to H5, the median averaged scores of desirability, feasibility and fit for each of the four stimuli was compared using the Mann–Whitney *U*-test. Results revealed that in the high condition of technologies, the participant population evaluated equally the desirability dimension (MdAC = 4.66, *n* = 112 vs. MdUST = 4.66, *n* = 81; *U* = 4248.50, *z* = −0.75, *p* > 0.1), the feasibility dimension (*U* = 3961.00, *z* = −1.51, *p* > 0.1) and the fit dimension (*U* = 4051.00, *z* = −1.27, *p* > 0.1). Similar results in the low condition of technologies were obtained in the desirability aspect (MdFID = 4.66, *n* = 110 vs. MdWBT = 4.66, *n* = 101; *U* = 5355.00, *z* = −0.45, *p* > 0.1), the feasibility (*U* = 5534.00, *z* = −0.05, *p* > 0.1) and in the fit aspect (*U* = 5313.00, *z* = −0.55, *p* > 0.1) of Opportunity Beliefs. Considering these results, the data were collapsed across each condition.

To test the effects of hypotheticality on the formation of beliefs about the desirability, feasibility and fit aspects of opportunities, a series of non-parametric ANCOVAs (Quade’s ANCOVAs) were conducted to examine the effects on the dependent variables, using technology awareness as a covariate.

Considering Feasibility beliefs on opportunities, respondents in the low condition (vs. high) of the level of hypotheticality, evaluated the feasibility dimension more favorably (MdFEASIB-High = 4.00, *n* = 193 vs. MdFEASIB-low = 4.50, *n* = 211; *F*(1,402) = 25.841, *p* < 0.001) (*α* = 0.85). These results are confirmed by a Kruskal-Wallis’s test pondering influences of high vs. low settings on feasibility beliefs [*χ*^2^(1, *N* = 404) = 20.49, *p* < 0.001], thus supporting H3.

Considering beliefs of Fit/Alignment on opportunities, respondents in the low condition evaluated this dimension more favorably, compared to respondents in the high condition (MdFIT-High = 4.66, *n* = 193 vs. MdFIT-low = 5.00, *n* = 211; *F*(1,402) = 6.91, *p* = 0.009) (*α* = 0.90). These results are confirmed by a Kruskal-Wallis’s test evaluating the effects of the high vs. low condition on fit beliefs [*χ*^2^(1, *N* = 404) = 5.30, *p* = 0.021], supporting H4.

Regarding Desirability beliefs in opportunities, results show insignificant differences in desirability concerns of the opportunity across the high-low conditions (MdDESIR-High = 4.66, *n* = 193 vs. MdDESIR-low = 4.66, *n* = 211; *F* (1,401) = 01.455, *p* > 0.1) (*α* = 0.81). These results are confirmed by a Kruskal-Wallis’s test considering effects of high vs. low conditions on desirability beliefs [*χ*^2^(1, *N* = 404) = 0.91, *p* > 0.1], supporting H5.

The results of Study 1 and Study 2 are summarized in Table 1.

**Table 1:** Summary of results of hypothesis test in Study 1 and Study 2.

Hypothesis test		Outcome
Hypothesis 1	Technologies perceived as more innovative (vs. less innovative) by entrepreneurs, will be sensed as more (vs. less) psychologically distant.	Supported
Hypothesis 2a	Degree of innovation in technologies (External Enabler) affects psychological distance, which in turn affects Opportunity Beliefs, such that psychological distance would mediate the relationship between the degree of innovation and Opportunity Beliefs.	Supported
Hypothesis 2b	Entrepreneurs will evaluate more favorable overall business Opportunity Beliefs when psychological distance (hypotheticality) is close, than when psychological distance (hypotheticality) is more distal.	Supported
Hypothesis 3	Entrepreneurs will evaluate more favorable the Feasibility dimension of Opportunity Beliefs when psychological distance (hypotheticality) is near, than when psychological (hypotheticality) distance is more distal.	Supported
Hypothesis 4	Entrepreneurs will evaluate more favorable the Fit/Alignment dimension of Opportunity Beliefs when psychological distance (hypotheticality) is near than when psychological distance (hypotheticality) is more distal.	Supported
Hypothesis 5	The difference in how entrepreneurs evaluate the Desirability dimension of Opportunity Beliefs when psychological distance is close compared to when it is more remote will be negligible.	Supported

#### 3.2.4 Discussion of Study 2

Study 2 provided empirical evidence of the impact of hypotheticality, as a component of psychological distance originating from the level of technological innovation, on the distinct facets of business Opportunity Beliefs. This examination was replicated using two different sets of technologies. As anticipated, the study uncovered that a low degree of technological innovation (compared to a high degree) led to more favorable assessments of Opportunity Beliefs. The results indicated that the subjective perception of distance (hypotheticality) served as a mediating factor in the relationship between the level of technological innovation in external enablers and Opportunity Beliefs. Additionally, participants showed uniform evaluations of the Desirability dimension of Opportunity Beliefs, but disparities emerged in the Feasibility and Fit/Alignment dimensions.

## 4 General discussion

How do perceptions of differences in the stimulus, the entrepreneurial enabler, influence the formation of Opportunity Beliefs? This study integrates elements of social psychology to develop a complementary framework for understanding how features of stimuli interact with cognitive processes in opportunity judgment. Variables at both the individual level (Forlani and Mullins, 2000; Simon et al., 2000; Mullins and Forlani, 2005; Uygur and Kim, 2016; Wood et al., 2017) and the stimulus level (Karahanna et al., 1999; Bhattacherjee and Premkumar, 2004; Hu and Reid, 2018) have been tested in previous studies to assess their impact on opportunity evaluation. However, prior research has overlooked the implicit connection between perception and opportunity evaluation, particularly in technology-based ideas, and how different degrees of innovation in new venture ideas impact Opportunity Beliefs. Through two experimental studies involving four different technologies that varied in their level of innovation, the results supported all proposed hypotheses. In this context, this research underscores the impact of hypotheticality as a dimension of psychological distance, as perceived subjectively in the External Enabler, particularly in relation to the level of innovation in technology-based business ideas, on the development of Opportunity Beliefs. This impact is evident in the assessment of the overall favorability of business Opportunity Beliefs, which subsequently affects various aspects of these beliefs: the feasibility and desirability of the business idea, and its alignment with the market.

Our findings in Study 1 and 2 suggest that entrepreneurs perceive technologies as more psychologically distant if they are perceived as more innovative, compared to less innovative technologies. This aligns with previous research indicating that the introduction of novel technological products serves as a catalyst for new business opportunities, evoking characteristics such as rarity, novelty, and change (Shane and Venkataraman, 2000). Variables such as the perceived familiarity of stimuli (Bhattacherjee and Premkumar, 2004), the level of change or novelty (Sääksjärvi and Hellén, 2019), interpretations of complexity (Karahanna et al., 1999), and uncertainty regarding potential outcomes (Hu and Reid, 2018) impact the development of Opportunity Beliefs. Our findings contribute additional insights to these concepts. This research indicates that the innovativeness of technologies leads to perceptions of unfamiliarity, unknowability, or inexperience, which, according to CLT, induces psychological distance from the observer’s perspective (*cf.* Trope et al., 2007). As new technological advancements often involve sophisticated scientific concepts and theoretical potentials, they are perceived as psychologically distant from individuals (Chiang et al., 2022). Therefore, the introduction of innovations in the form of new functionalities and improvements compared to existing alternatives can significantly influence how they are perceived in terms of psychological distance, as they create perceptions of unfamiliarity and lack of experience with them (Bhattacherjee and Premkumar, 2004).

Our Study 2 results suggest that psychological distance acts as a mediating factor between the perceived level of innovation and Opportunity Beliefs. This finding enhances prior research on Opportunity Evaluations (e.g., Davidsson, 2015; Kimjeon and Davidsson, 2022) by introducing an intermediate cognitive process into the understanding of the phenomenon. Specifically, our results confirm the hypothesized indirect influence of Opportunity Beliefs through psychological distance. This is in line with previous research indicating that other dimensions of psychological distance, as in the case of temporal distance (Tumasjan et al., 2013), serve as mediators with Opportunity Beliefs. This finding is significant as it aligns with the concept of a two-phase process in opportunity evaluation (Andrade-Valbuena and Torres, 2018), wherein the perceptual process is crucial. This process provides a novel framework of information from which entrepreneurs formulate and assess assumptions regarding the relationship between the demand and supply aspects of an opportunity, as well as how individuals can benefit from its exploitation (Baron and Ensley, 2006; Mitchell et al., 2017). Our proposed mediated model resembles previous theorizations, such as the first- and third-person opportunity evaluation process described by McMullen and Shepherd (2006). This model integrates the primary concerns of value creation and value appropriation during the stages preceding entrepreneurial action. In contrast, our model suggests a two steps perception-judgment process mediated by psychological distance.

It is expected that entrepreneurs form more favorable overall business Opportunity Beliefs when psychological distance (hypotheticality) is perceived as close, compared to when it is perceived as more distant. This aligns with earlier studies suggesting that the speculative nature of business concepts, which vary in their levels of technological innovation, begins to parallel gambling scenarios (see Barney, 1997; Danneels, 2004). Our results further elucidate these notions. Consistent with previous research (Chen et al., 2018; Mount et al., 2021), our study indicates that for ideas based on disruptive technologies, the likelihood of technological adoption and commercial success in a business context resembles scenarios that are less credible, harder to envision, and thus more hypothetical. Conversely, less innovative technologies in a business context lead to scenarios that are typically seen as more concrete, and thus are more easily associated with tangible products or solutions, increasing their perceived probability of occurrence (Akdim et al., 2023). Therefore, the premise underlying the concept of Abstractness, which suggests that higher (vs. lower) probabilities of realization enhance (vs. diminish) entrepreneurs’ overall Opportunity Beliefs, as proposed by Chen et al. (2018), is supported and maintained for less innovative (vs. more innovative) technologies, as they are perceived as less (vs. more) abstract.

According to previous research, opportunity beliefs are influenced by perceived uncertainty regarding the Feasibility, Desirability, and market Fit of an entrepreneurial idea (Grégoire et al., 2009; Grégoire and Shepherd, 2012). Based on our hypothesis, we found that lower levels of perceived innovation positively affect the feasibility dimension of opportunity beliefs, likely by reducing the uncertainty surrounding an entrepreneurial idea through perceptions of hypotheticality. Entrepreneurs are more convinced of a business idea’s feasibility when they perceive lower levels of innovation, thereby reducing their psychological distance. Additionally, this study aligns with research suggesting a temporal distance relationship with the feasibility dimension of opportunity beliefs (Tumasjan et al., 2013). When an opportunity can be exploited in the near future rather than in the distant future, entrepreneurs are more influenced by the feasibility characteristics of the opportunity than by its desirability characteristics (Tumasjan et al., 2013). These results are also consistent with Construal Level Theory (CLT), as the value associated with feasibility (a low-level interpretation) tends to be more pronounced at closer psychological distances, particularly, in this research, at lower levels of perceived hypotheticality (Liberman and Trope, 1998). Therefore, these results demonstrate that the inherent characteristics of a business opportunity lead entrepreneurs to perceive differences in the feasibility of a business opportunity.

Consistent with our hypothesis we find that entrepreneurs will evaluate the Fit/Alignment dimension of Opportunity Beliefs more favorably when psychological distance (hypotheticality) is perceived as near, compared to when it is perceived as more distant. This finding contributes additional insights to previous research, in the link between the stimulus and the fit/alignment dimension of Opportunity Beliefs. For instance, Chen et al. (2018) suggest that the entrepreneur’s perspective on a new business is positively associated with the construal level of entrepreneurial activity, like attracting customers while competing with other companies, across the spectrum from considering the socioeconomic aspects to actually engaging in action. Our findings indicate that when entrepreneurs encounter business ideas with reduced hypotheticality, they are more likely to adopt a concrete and detailed perspective. In this view, they perceive the alignment between market needs and demands as an achievable goal. Therefore, the evidence from this sample provides further insight into these concepts.

Finally, consistent with our hypothesis, the findings indicate that the difference in how entrepreneurs evaluate the Desirability dimension of Opportunity Beliefs between scenarios of high versus low hypotheticality (derived from its high level of innovation in tech-based business ideas) is not significant. In other words, as the likelihood of a business idea materializing increases, entrepreneurs evaluate the opportunity that is more achievable (low level of hypotheticality) by considering both efforts and rewards. In contrast, when the likelihood of a business idea materializing is lower (high level of hypotheticality), entrepreneurs focus solely on the attractiveness of the outcome. Therefore, no significant difference is expected in how desirable the opportunity is between these scenarios. These results align with Construal Level Theory (CLT), which suggests that the increase in probability affects the weighting of means-related features in decisions but does not affect the weighting of ends-related features (Todorov et al., 2007; see also Liviatan et al., 2008). These results differ from those of Tumasjan et al. (2013), who find that when an opportunity can be taken advantage of in the distant future (as opposed to the near future), entrepreneurs are more influenced by desirability considerations rather than feasibility considerations, compared to feasibility considerations over desirability considerations (see H1 in such study). As suggested by Todorov et al., (2007, p. 479), means-related and ends-related features are weighted differently in decisions based on probability, with desirability and feasibility reasons playing unique roles at various probability levels. Specifically, when probability is high, both types of reasons are important for decision-making, but when probability is low, only ends-related reasons matter. Thus, the premise that the dimension of temporal distance differs from the dimension of hypotheticality, as proposed by Todorov et al., (2007) and Liviatan et al. (2008), is supported and maintained in this study. This study specifically examined tech-based business ideas with high levels of innovation. Consequently, these contextual differences lead to distinct evaluations of opportunity beliefs and decision-making processes among entrepreneurs.

## 5 Contributions to theory and practice

By integrating elements of social psychology, this research is positioned to offer valuable contributions to the entrepreneurship literature and the theory of psychological distance, with a focus on subjective perceptions of External Enablers and their role in shaping business Opportunity Beliefs. This study makes significant contributions to both theory and practice, which will be discussed below.

First and foremost, this study highlights the involvement of technological innovation in the formation of hypothetical judgments, which in turn affect cognitive evaluations of their suitability as a foundation for new economic ventures. In this sense, this work provides relevant contributions to the research streams initiated by Grégoire and Shepherd (2012), Davidsson (2015), von Briel et al. (2018), and Kimjeon and Davidsson (2022) which are based on the differences among technological opportunities seen as External Enablers. Along this same line, Shepherd et al. (2019) and Shepherd (2015) have recommended research focused on how opportunities emerge and the role that different mindsets and cognitive orientations play in the initiation of entrepreneurial endeavors. These studies help fill those gaps by building bridges, testing, and documenting relationships with social psychology, and basing research on psychological distance derived from Construal Level Theory. In particular, this study demonstrates the differential impact of hypotheticality on the weighting of desirability and feasibility features in the evaluation of opportunities and decision-making.

Secondly, it extends the research trajectory initiated by Grégoire et al. (2009) and Grégoire and Shepherd (2012), which emphasizes the distinctions among technological opportunities. Building on this, (Shepherd, 2015; Shepherd et al., 2019) have advocated for research that delves into how opportunities emerge and the role that different mindsets play in the initiation of entrepreneurial ventures. We believe our work effectively addresses these gaps by establishing connections, testing relationships, and documenting findings within the framework of Construal Level Theory from the realm of social psychology.

Thirdly, our results align with previous findings from Tumasjan et al. (2013) by presenting a context rooted in perceptions of technological innovations, where concerns regarding feasibility, fit and desirability in business opportunities are differentially influenced by hypotheticality. Particularly, the findings of this study contribute significantly to the understanding of how hypotheticality influences the evaluation of opportunities. By demonstrating the differential impact of hypotheticality on the weighting of desirability and feasibility features, our research highlights the importance of considering psychological distance in entrepreneurial decision-making. In this line of thought, our study found that the likelihood of a business idea materializing affects how entrepreneurs evaluate the desirability feasibility and fit of opportunities. Specifically, as the probability of success increases, entrepreneurs tend to favor opportunities that are easier to achieve, even if they are less attractive. This underscores the critical role of psychological distance in shaping entrepreneurial perceptions and opportunity beliefs. This insight enhances the theoretical framework of Construal Level Theory (CLT) and its application in entrepreneurship research.

For instance, considering the opening case about Blockbuster vs. Netflix through the lens of Construal Level Theory (CLT), one can observe specific aspects that are uniquely illuminated by this theory. CLT highlights how psychological distance influences perception, which might remain less apparent in other theoretical frameworks. These divergences in perception regarding technology or business ideas rooted in technology lead to altered perceptions of products, clients, and locations. In this sense, CLT helps explain the differences in Opportunity Beliefs between both CEOs through the perspective of psychological distance. Blockbuster’s CEO at the time, John Antioco, viewed online streaming as distant from the company’s physical rental store model. This perception caused him to undervalue the opportunity focused on online streaming. In contrast, Netflix’s CEO, Reed Hastings, perceived the streaming opportunity as closer and more relevant to Netflix’s business. His experience as the founder of software development and troubleshooting ventures, as well as his presence on the board of directors for several tech companies, likely influenced this perception, and therefore, his evaluation of this opportunity (i.e., Opportunity Beliefs).

Lastly, this research adds value by examining the mediating role of psychological distance in the relationship between the degree of innovation in technologies and Opportunity Beliefs. By exploring this mediation, researchers can gain a deeper understanding of the cognitive processes that underlie the formation of Opportunity Beliefs. This analysis helps elucidate the mechanism through which innovation influences Opportunity Beliefs.

From a practical perspective, the study’s results emphasize the critical role of perception and interpretation of external conditions as the foundation for business opportunities. Entrepreneurs regularly face opportunities that may involve discerning differences in the circumstances giving rise to their business prospects. Understanding these perceptual and cognitive processes can inform better decision-making strategies, training programs, and policies aimed at fostering entrepreneurial success. Entrepreneurs can enhance their decision-making capabilities by better assessing opportunities through an in-depth understanding of how psychological distance influences their evaluations of desirability and feasibility. This means that entrepreneurs can make more informed and strategic choices, which could lead to higher success rates in their ventures. Additionally, entrepreneurship training programs can greatly benefit from incorporating these findings on psychological distance. By doing so, these programs can help entrepreneurs develop more accurate and balanced Opportunity Beliefs, equipping them with the cognitive tools necessary to navigate complex business environments effectively.

For policymakers, the insights from this research suggest designing initiatives that consider the cognitive factors affecting entrepreneurial decisions. Such initiatives could provide targeted support to entrepreneurs, fostering an environment that encourages the development of new ventures. This could include policy measures that reduce perceived barriers to entry or that provide resources tailored to the specific cognitive needs of entrepreneurs.

Furthermore, by highlighting the role of innovation in shaping Opportunity Beliefs, this research encourages a more nuanced approach to evaluating technological opportunities in entrepreneurial contexts. Entrepreneurs and investors can use this understanding to better gauge the potential of innovative technologies and to allocate resources more effectively. This perspective can lead to more sustainable and impactful entrepreneurial endeavors.

## 6 Conclusion

In conclusion, this study sheds light on the intricate dynamics between psychological distance and the evaluation of business opportunities. The findings indicate that cognitive factors such as hypotheticality significantly impact how entrepreneurs assess the desirability, feasibility, and fit of potential business ventures. This observation suggests that entrepreneurs’ perceptions and judgments about opportunities are deeply influenced by their psychological distance from the perceived opportunity. Consequently, it is inferred that reducing psychological distance can enhance the perceived feasibility and desirability of a business idea, thereby influencing entrepreneurial decision-making processes.

This research responds to different calls to investigate the relationships between various dimensions of psychological distance in uncertain environments (Tumasjan et al., 2013; Chen et al., 2018; Maiella et al., 2020), with a specific focus on the cognitive factors influencing Opportunity Beliefs. Our findings demonstrate how cognitive factors such as hypotheticality impact the evaluation of business opportunities. By highlighting the role of hypotheticality, this study contributes to a deeper understanding of the cognitive processes underlying opportunity evaluation.

Additionally, our proposal of an alternative model bridging theoretical domains, as exemplified by the Construal Level Theory (CLT), enhances our understanding of how business opportunity beliefs and opportunity confidence develop. This contributes to establishing connections and explanatory mechanisms that facilitate the comprehension of opportunity judgments. The application of CLT in this context provides a robust framework for examining how psychological distance influences entrepreneurial cognition and behavior.

Moreover, we employed non-parametric ANCOVAs to test the effects of hypotheticality on the formation of beliefs related to desirability, feasibility, and fit aspects of opportunities, using technology awareness as a control variable. This methodological approach ensured a comprehensive analysis of our findings and reinforced the validity of our results. The use of technology awareness as a control variable further emphasizes the importance of contextual factors in shaping entrepreneurial perceptions.

## 7 Limitations and future research

One limitation of this study, arising from the research settings, is the inability to incorporate other crucial variables that may have significant impacts. For example, the evaluation of psychological distance might yield conflicting values related to high-level and low-level construals, particularly in scenarios where cognitive tendencies, such as analytic vs. holistic thinking styles, predominate, as observed in consumer contexts (Monga and John, 2007). Similarly, when considering technological cognitive styles, variables like technological reflectiveness (Andrade-Valbuena and Torres, 2018), technological optimism (Parasuraman, 2000), technological self-efficacy (Compeau and Higgins, 1995), technological anxiety, and social influences on technology adoption (Venkatesh et al., 2003) could be taken into account in future research.

Additionally, this study is specifically focused on technological-based business ideas as stimulus. Future research could explore different types of stimuli. For example, while extensive prior research has examined aspects related to addressing customers’ problems and needs (i.e., Shepherd and DeTienne, 2005; Prandelli et al., 2016), how entrepreneurs perceive such needs and how the various dimensions of psychological distance affect the New Venture Ideation process have not been explored. This opens the door to various research directions, from international new venture formation (Nowiński and Rialp, 2016) to opportunity/necessity-motivated entrepreneurship (Nikolaev et al., 2018) and ethical entrepreneurship (Yitshaki and Kropp, 2017).

Moreover, other aspects of Stimulus, such as gender or stereotypical information (Gupta et al., 2013, 2014); implicitness and availability of information (Smith et al., 2009), or information that could potentially bias decisions, such as potential harm to the natural environment (Shepherd et al., 2012), have not been examined in the context of perceptions of psychological distance. These aspects present opportunities for further research building upon existing lines of inquiry.

Discussing the possible factors that were not controlled for and how they might influence the results in this study, several aspects need consideration. Individual differences in cognitive styles, such as analytical versus holistic thinking, can significantly impact how participants perceive and evaluate opportunities. Participants’ prior experience with technology and their level of technological savviness were also not controlled for, which could lead to different perceptions and evaluations of technological opportunities. Cultural and social background differences among participants were not accounted for, and these can shape how individuals perceive risks and opportunities. Additionally, the specific market conditions and economic environments in which the participants were operating were not considered, potentially affecting the perceived feasibility and desirability of business opportunities. The psychological state and mood of the participants at the time of the study, such as stress or optimism, could also influence decision-making processes. External influences and the availability of information, like media reports or advice from peers, were not controlled, which can shape participants’ perceptions. Furthermore, the timing of the study in relation to significant technological advancements or market changes was not taken into account, potentially impacting the participants’ evaluations of opportunities.

Based on our findings, future research could investigate several key areas. One important area is the examination of diverse stimuli, particularly how different types of business ideas, such as social enterprises and non-technological ventures, influence opportunity evaluation. This research could include exploring how social enterprises or environmentally focused ventures are perceived differently compared to tech-based ventures and how these perceptions influence decision-making processes.

Another area of interest is cognitive styles. Future studies could explore the impact of various cognitive styles, such as analytical versus holistic thinking, on the perception of psychological distance and opportunity evaluation. Research could delve into how entrepreneurs with different cognitive styles process information and assess opportunities, potentially revealing new insights into cognitive diversity in entrepreneurship.

Technological influences also warrant further investigation. Research could focus on the role of technological optimism, self-efficacy, and anxiety in shaping entrepreneurial decisions. Understanding how these factors influence the willingness to adopt new technologies and how they affect the perception of feasibility and desirability in different technological contexts could provide valuable insights for supporting entrepreneurial ventures.

Finally, studying social and environmental factors is crucial. Future research could examine how social influences and concerns about environmental impact affect the perception of business opportunities. This line of research could explore how social norms, peer influence, and ethical considerations shape entrepreneurial judgments and the prioritization of sustainable business practices. These investigations could lead to a deeper understanding of how external social and environmental factors impact entrepreneurial decision-making and opportunity evaluation.

## Data availability statement

The raw data supporting the conclusions of this article will be made available by the authors, without undue reservation.

## Ethics statement

Ethical review and approval was not required for the study on human participants in accordance with the local legislation and institutional requirements. The participants provided their written informed consent to participate in this study.

## Author contributions

NA-V: Writing – review & editing, Writing – original draft, Visualization, Validation, Supervision, Software, Resources, Project administration, Methodology, Investigation, Funding acquisition, Formal analysis, Data curation, Conceptualization. SS: Writing – review & editing, Writing – original draft, Visualization, Validation, Supervision, Software, Resources, Project administration, Methodology, Investigation, Funding acquisition, Formal analysis, Data curation, Conceptualization. CJ: Writing – review & editing, Writing – original draft, Visualization, Validation, Supervision, Software, Resources, Project administration, Methodology, Investigation, Funding acquisition, Formal analysis, Data curation, Conceptualization.
