# Supplementary material for: Perception of hypotheticality in technology-based business ideas: effects on Opportunity Beliefs from a Construal Level Theory perspective
Source: Front Psychol. 2024 Jun 7;15:1404726. doi: 10.3389/fpsyg.2024.1404726 (PMC11191069; doi:10.3389/fpsyg.2024.1404726)
Supplement: Supplementary file 1 [file Data_Sheet_1.PDF]

Supplementary Material

1 Vignettes: Example descriptions of technologies used in experiments

Technology 1 (Low condition): WATERPROOF BREATHABLE TEXTILES

Wearing **Waterproof Breathable Textiles (WBTs)** seems like something only sporting people could love. But waterproof breathable fabrics are showing up in places other than playing fields and sports centers.

From sleeping to jogging, the body provides cooling through perspiration, creating a microclimate inside the clothing which makes it uncomfortable to wear. As opposed to water-repellent fabric, which only delays the penetration of water, **WBTs** are designed to diffuse body heat through the fabric and still offer protection from wind and rain.

WBT applications can challenge the apparel function barrier. Fabrics that repulse many harmful chemical, biological and physical agents, while permitting the effective transmission of moisture vapor from inside to the outside atmosphere are now feasible. Applications of these technologies have been tested in the packaging of pharmaceutical products, helping them to retain their coolness, while protecting them from environmental agents.

WBTs are expected to witness a rapid growth on account of their ability to effectively deal with the problems and needs of people's everyday lives. This increasing usage in a countless range of applications suggests a fertile ground that an imaginative person on the look-out for opportunities could take advantage of.

Technology 1 (High condition): ULTRA-SMART TEXTILES

Auto-adjusting and auto-drying jackets and other '*intelligent garments*' that seemed only possible in the movies, are a reality, thanks to important innovations in **Ultra-Smart Textiles (USTs)**.

USTs are fabrics that respond to different inputs, reacting and adapting to the user's behavior in different circumstances and inputs. A **UST** essentially consists of a unit that works like the brain, with cognition, reasoning, but also with activating capacities. This has been possible thanks to a successful marriage with other branches of innovation such as nanotechnology, miniaturized electronic components, and sensors and actuators.

UST applications can challenge the limitations of our biology. Lightweight suits that leverage our physical power or speed, while helping to prevent injuries are feasible. Applications of this technology have been tested, helping the elderly in their mobility by tracking postures and movements, while assisting them when sitting up, standing upright and raising their arms.

USTs are expected to witness a rapid growth on account of their ability to effectively deal with the problems and needs of people's everyday lives. This increasing usage in a countless range of

applications suggests a fertile ground that an imaginative person on the look-out for opportunities could take advantage of.
